# Mining Social Media to Identify Heat Waves

**DOI:** 10.3390/ijerph16050762

**Published:** 2019-03-02

**Authors:** Francesca Cecinati, Tom Matthews, Sukumar Natarajan, Nick McCullen, David Coley

**Affiliations:** 1 Department of Architecture and Civil Engineering, University of Bath, Bath BA2 7AY, UK; s.natarajan@bath.ac.uk (S.N.); nm268@bath.ac.uk (N.M.); dac33@bath.ac.uk (D.C.); 2 Department of Geography and Environment, Loughborough University, Loughborough, Leicestershire LE11 3TU, UK; t.matthews@lboro.ac.uk

**Keywords:** heatwave, heatwave definition, Twitter mining, social media

## Abstract

Heat waves are one of the deadliest of natural hazards and their frequency and intensity will likely increase as the climate continues to warm. A challenge in studying these phenomena is the lack of a universally accepted quantitative definition that captures both temperature anomalies and associated mortality. We test the hypothesis that social media mining can be used to identify heat wave mortality. Applying the approach to India, we find that the number of heat-related tweets correlates with heat-related mortality much better than traditional climate-based indicators, especially at larger scales, which identify many heat wave days that do not lead to excess mortality. We conclude that social media based heat wave identification can complement climatic data and can be used to: (1) study heat wave impacts at large scales or in developing countries, where mortality data are difficult to obtain and uncertain, and (2) to track dangerous heat wave events in real time.

## 1. Introduction

Heat waves are amongst the deadliest of natural hazards [1,2,3]. The European heat wave during the summer of 2003 resulted in 22,000 to 70,000 excess deaths [4], while the Russian heat wave of 2010 caused 56,000 [5]. The duration, frequency, intensity and extent of heat waves has also increased since the end of 20th century, and climate models project this will continue with global warming in the near future [6,7,8]. Heat waves are also expected to be exacerbated by other global trends like increasing urbanization [9,10,11], and will impact human health, as well as ecosystems, economy, electricity and water consumption [12].

To ensure consistent and comparable studies across institutions, countries, and disciplines, a common definition of a heat wave is necessary. The Glossary of Meteorology (GoM) formally defines a heat wave as “a period of abnormally and uncomfortably hot and usually humid weather” [13]. Whilst a widely accepted definition, it is ambiguous in the absence of quantitative guidelines. This is reflected in the great range of heat wave definitions employed in the literature, which can differ in terms of metrics (e.g., maximum temperature, minimum temperature, mean temperature, apparent temperature), selected threshold that needs to be exceeded (e.g., an absolute threshold, a percentile, or a an anomaly from the climatological mean), or duration of the exceptional conditions (usually ranging from 2 to 5 days at least) [14]. Numerous researchers have attempted to organise the proposed definitions [14,15,16,17,18,19], but a universal solution remains elusive. One reason is that, looking at the GoM definition, it is relatively easy to quantify the abnormality of temperature and humidity, but the quantification of discomfort for the population is a much harder problem.

Critical thresholds to identify heat waves are often selected through regression analysis of the temperature-mortality relationship [19,20,21,22,23,24,25,26,27,28,29,30,31,32]. However, there are practical challenges that limit this approach as a universal solution. For example, the relationship between temperature metrics and mortality has been shown to vary geographically, with socioeconomic or demographic factors, mortality and morbidity data are sensitive, not widely available, and challenging to obtain, and the death toll from heat events can be significantly under- or mis-reported (particularly in developing countries).

This work proposes a very different impact-led approach to identify heat waves using Twitter. Billions of people now use social media to discuss significant world events, and Twitter, with its concise 140-character limit, is regarded as a de-facto tool for mining “what’s happening now” [33]. This recognition has seen researchers use Twitter for event detection in the context of breaking news [34,35], music events [36], forest fires [37], earthquakes [38], and flu outbreaks [39]. In previous studies, Twitter has been used to identify heat-risk, but only at local scale, for a short time span and without considering impact on population mortality [40]. Our study is the first to use the widespread availability of Twitter data at large spatial and temporal scales, thus enabling to compare time series with heat-related mortality.

Our study specifically considers Twitter posts (tweets) containing heat wave related phrases, to identify heat waves in India. The choice of India as a case study is due to: (1) India being a heavily populated country (1.34 billion in 2016) [41]; (2) extreme heat is a very prominent (and growing) natural hazard in the country [22,42,43,44,45]; (3) India is a developing country and access to heat-related mortality data is difficult and uncertain, due to significant under-reporting of death or mis-attribution of causes; and (4) English is widely used for social media.

The first question we address is whether Twitter data are better than climate indicators at detecting heat effects on public health, specifically looking at heat-related mortality. After confirming that Twitter data is strongly correlated with heat-related mortality, we examine the differences in heat-wave identification between Twitter and climate data. This step is pursued because we anticipate that Twitter cannot be used for forecasting (and hence warning), which must be informed by meteorological indicators. Hence, a thorough understanding of their similarities and differences is required.

## 2. Materials and Methods

Our analysis is divided in four parts. At first, we obtain and analyses Twitter data related to heat waves in India. Subsequently, we compare these data with heat-related mortality data, as well as climatic indicators. We repeat this analysis for the two Indian states most affected by heat waves in the study period, to demonstrate the validity of the approach at regional scale as well. Finally, we compare the Twitter data and common heat wave definitions.

### 2.1. Datasets

#### 2.1.1. Twitter Data

We download and count the tweets containing the phrases “heat wave India” and “heatwave India” between 1 January 2010 and 31 December 2017 and the dataset is fully available [46]. To overcome the Twitter API limitations in the number of downloadable tweets and the time span that can be covered, we use a “scraping” algorithm contained in the GetOldTweets-Python package that accesses the Twitter platform, searches for the desired phrases, scrolls down and downloads all the results [47]. The drawback of the methodology is that it cannot access the tweet geolocation. For this reason we include the country name in the search phrase. These data are likely to only cover a small fraction of the tweets related to extreme temperatures within the country, as many people will presumably not include “India” in the tweet, or may use different, possibly more expressive, wording, but by using a single phrase we ensure consistency. The selected keyword is less ambiguous than other common expressions like “hot”. We can assume that the use of a subset of all tweets referring to Indian heat waves is representative of the full sample and filters only the most significant events, which are likely the ones affecting population health. This approach hence provides a conservative test of our hypothesis.

**Figure 1:**
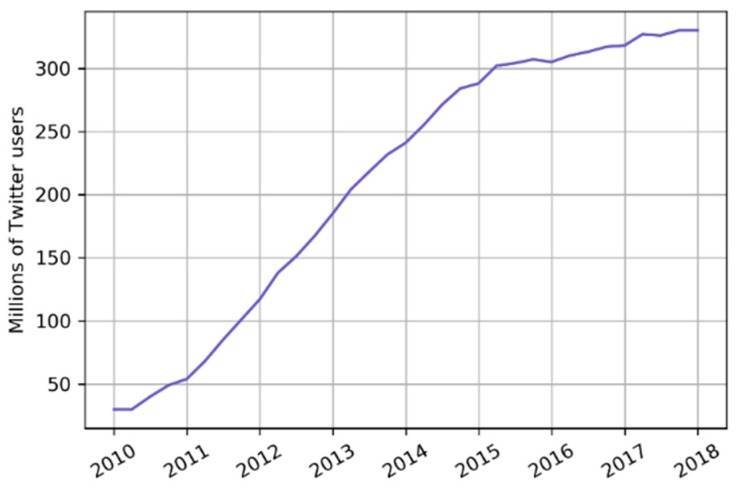
The figure shows the number of Twitter users active globally.

The number of daily tweets about heat waves in India is then normalised to account for the varying number of active Twitter users over time. The global number of active Twitter users per quarter, between 2010 and 2017, was accessed through the Statista website that collected and organised publicly available data from Twitter [48]. The dataset is shown in Figure 1. For finer temporal resolutions, a linear interpolation is used.

Finally, to compare Twitter data to heat wave definitions, the time series of daily tweets about heat waves in India is converted to binary using a 2-tweet threshold: i.e., we consider that a heat wave has occurred if we count at least two tweets per day (other thresholds were tested as well, but resulted in lower correlation with climate-based heat wave definitions).

#### 2.1.2. Climate Data

To calculate climatic indicators and heat wave definitions we need temperature and relative humidity data for India. Station data at a daily temporal resolution is not easy to access in India, spatial coverage is not always sufficient, records length variable, and quality inconsistent; thus we use the European Centre for Medium-range Weather Forecasts (ECMWF) ERA-interim re-analysis, available between 1979 and 2017 at 0.75° lat/lon and 6 h resolution through the ECMWF API service [49]. ERA-interim provides air temperature at 2 m and relative humidity is derived from air and dew point temperatures [50]:

(1) $$\begin{array}{c} R=100\;\left(10\cdot \exp\left(\frac{17.625\cdot T_{d}}{243.04+T_{d}}\right)\right)/\left(10\cdot \exp\left(\frac{17.625\cdot T}{243.04+T}\right)\right)\;\; \end{array}$$

where R is the relative humidity in [%], $T_{d}$ is the dew point temperature in [°C], and T is the air temperature [°C]. Data between 1980 and 2009 are used to obtain the 30-year climatological statistics, while data between 2010 and 2017 are used for the analysis. Mean and percentiles are calculated for each pixel, over the whole 30-year time series, without differentiating the day of the year or the season.

#### 2.1.3. Population Data

We hypothesise that the mortality impact of a heat wave is proportional to the number of affected people. To consider this aspect, temperature, Heat Index and Excess Heat Factor (better defined in Section 2.4) scaled with population data are also considered and compared to mortality datasets. Population data is obtained from the Gridded Population of the World dataset, adjusted according to the United Nation World Population Prospect (UN WPP-Adjusted Population Density, v 4.10) [51]. The dataset refers to 2015 and is downloaded at a resolution of 0.25° lat/lon, subsequently re-gridded on the 0.75° grid of the ERA-interim climatic data.

#### 2.1.4. Heat-Wave Related Mortality

We use three heat wave excess mortality datasets to account for the uncertainty in heat-related mortality data: (1) the international EM-DAT database [52], (2) the official data from the National Disaster Management Authority (NDMA) [53] and (3) the data manually extracted from the seasonal and annual reports from India Meteorological Department (IMD) retrieved from the MAUSAM: Quarterly Journal of Meteorology, Hydrology, and Geophysics repository. All the sources provide annual data and the IMD reports also provide data at monthly resolution, which is then used to reinforce the analysis at a finer temporal scale.

### 2.2. Selection of Climatic Indicators

The correlation between mortality data, number of tweets and a set of nine climatic indicators is considered. As mortality data is only available at yearly scale, yearly climatic indicators are selected as well. The selected indicators are chosen to cover a variety of metrics (maximum daily temperature, mean daily temperature, Heat Index, Excess Heat Factor and affected population) and different statistics (absolute maximum or threshold exceedance):

- Maximum mean daily temperature reached in the year ($T_{mM}$)
- Maximum maximum daily temperature reached in the year ($T_{MM}$)
- Maximum heat index reached in the year ($HI_{M}$)
- Maximum excess heat factor reached in the year ($EHF_{M}$)
- Maximum difference between the mean daily temperature and the 95th percentile of mean daily temperature reached in the year ($T_{diffM}$)
- Maximum difference between the heat index and the 95th percentile of heat index reached in the year ($HI_{diffM}$)
- Maximum difference between the mean daily temperature and the 95th percentile of mean daily temperature scaled by the affected population ($T_{diffMpop}$)
- Maximum difference between the heat index and the 95th percentile of heat index scaled by the affected population ($HI_{diffMpop}$)
- Maximum Excess Heat Factor scaled by the affected population ($EHF_{Mpop}$)

### 2.3. Selection of Regions Most Affected by Heat Waves

Most of the data in this work is not available at regional scale. However, the IMD reports about the weather offer some information on the distribution of the heat-related deaths throughout the different Indian states.

**Figure 2:**
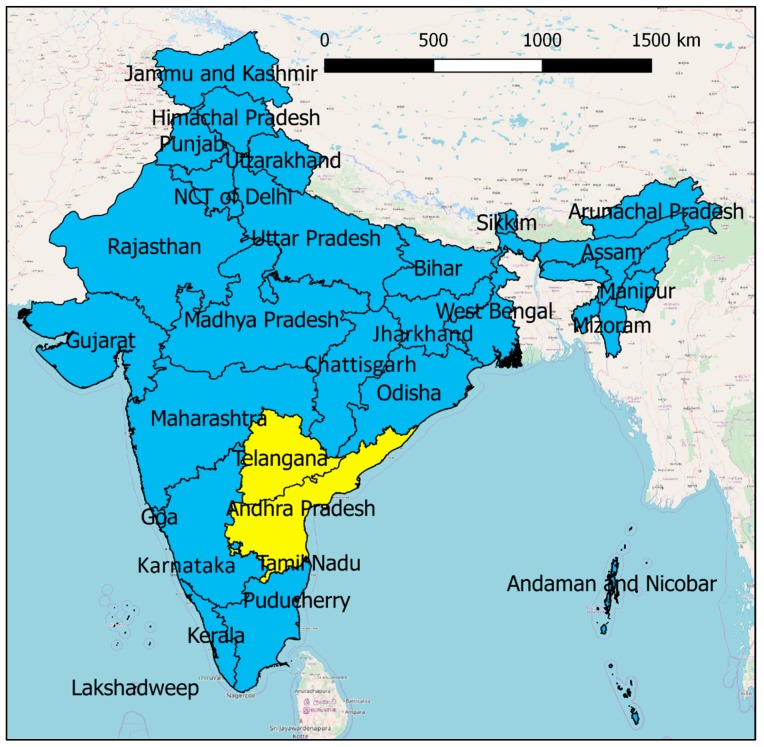
Indian states. The two selected for the regional analysis being the most affected by heat waves in the study period, Andhra Pradesh and Telangana, are highlighted in the figure.

A summary is shown in Table 1, and the position of the selected states and all the other states is shown in Figure 2. We cannot repeat the analysis for all the Indian states, thus we select the two most affected ones in the study period (IMD reports available only up to 2015). It must be noted that the IMD source of mortality data is the most uncertain one, thus we decided to test the approach on only two states, as the uncertainty becomes too large for smaller mortality numbers. According to the table, we select the states of Andhra Pradesh and Telangana. Subsequently, we download tweets containing the strings “heat wave Andhra Pradesh”, “heatwave Andhra Pradesh”, “heat wave Telangana”, and “heatwave Telangana”.

### 2.4. Selection of Heat Wave Definitions

Heat wave definitions, contrarily to climatic indicators, only return a binary outcome (heat wave/no heat wave) calculated on the basis of climatic data. Here some of the most common are selected.

#### 2.4.1. The Official IMD Definition (IMD)

The IMD adopts a heat wave definition that uses a mix of absolute and relative thresholds. A heat wave is declared in any Indian location if at least one of the following three conditions occurs [43]:

(2) $$\begin{array}{c} \left(T_{M}>45\;{}^{\circ}C\right)\vee (T_{M}>\bar{T_{M}}+4\;{}^{\circ}C|\;\bar{T_{M}}>40\;{}^{\circ}C)\vee (T_{M}>\bar{T_{M}}+5\;{}^{\circ}C\;|\;\bar{T_{M}}<40\;{}^{\circ}C) \end{array}$$

where $T_{M}$ is the maximum daily temperature, $\bar{T_{M}}$ is the average of the maximum daily temperature over the 30-year reference period, and the symbol ∨ represent the logical operation “or”. No minimum duration is set.

#### 2.4.2. The 95th Percentile for 2+ Days of Daily Mean Temperature (T95)

Heat wave definitions based on the exceedance of a relative threshold for a certain duration are very popular and several combinations of relative thresholds and durations are tested in literature. Here we select a threshold equal to the 95th percentile of daily mean temperatures for a duration of 2 or more days, as it is popular in literature [20,25,27,54].

#### 2.4.3. The 95th Percentile for 2+ Days of Daily Heat Index (HI95)

The Heat Index (HI) has been used for a long time, especially in the USA, and is also known as apparent temperature. Although many definitions exist, we use an empirical equation that describes HI[–] as function of air temperature T[°F] and relative humidity R[%] [55]:

(3) $$\begin{array}{c} HI=-42.38+2.05\cdot T+10.14\cdot R-0.22\cdot TR-6.84\cdot 10^{-3}T^{2}-5.48\cdot 10^{-2}R^{2}+\\ +1.23\cdot 10^{-3}T^{2}R+8.53\cdot 10^{-4}TR^{2}-1.99\cdot 10^{-6}T^{2}R^{2} \end{array}$$

A heat wave is defined as a period of at least two days in which the mean daily heat index exceeds the 95th percentile of mean daily heat index [56].

#### 2.4.4. The Excess Heat Factor (EHF)

The Excess Heat Factor (EHF) has been introduced relatively recently [3,57], but has grown in popularity [22,58,59,60]. The EHF is the combination of two components: the Significance Excess Heat Index ($EHI_{sig}$) represents the 3-day average temperature to capture unusually high heat that is not sufficiently discharged overnight, while the Acclimatisation Excess Heat Index ($EHI_{accl}$) represents the 30-day average temperature, to consider people adaptation to previous climatic conditions:

(4) $$\begin{array}{c} EHF=\max\left(0,EHI_{sig}\right)\cdot \max\left(1,\;EHI_{accl}\right) \end{array}$$

(5) $$EHI_{sig}=\bar{T_{3d}}-T_{95}$$

(6) $$\begin{array}{c} EHI_{accl}=\bar{T_{3d}}-\bar{T_{30d}} \end{array}$$

where $\bar{T_{3d}}$ is the average of the mean daily temperature over three days, $T_{95}$ is the 95th percentile of mean daily temperatures and $\bar{T_{30d}}$ is the 30-day average of mean daily temperatures. A heat wave is defined as a period of any length when the EHF is positive.

### 2.5. Evaluation of Climatic Heat Wave Definitions

As heat wave definitions are binary, we select five binary skill scores to compare the heat wave definitions to the Twitter data. The binary skill scores are described in Equations (7)–(11). They are based on the following definitions: a is the number of heat wave days both identified by Twitter and by the considered climatic definition; b is the number of heat wave days occurred according to Twitter, but not captured by the climatic definition; c are the number of heat wave days that are not identified by Twitter, but that the climatic definition considers as heat wave days; d is the number of days that are not considered as heat wave days by neither Twitter nor the climatic definition:

(7) $$\begin{array}{c} Percentage\;Correct=\frac{a+d}{a+b+c+d} \end{array}$$

(8) $$\begin{array}{c} Hit\;Rate=\frac{a}{a+c} \end{array}$$

(9) $$\begin{array}{c} Miss\;Rate=\frac{c}{a+c} \end{array}$$

(10) $$\begin{array}{c} False\;Alarm\;Rate=\frac{b}{b+d} \end{array}$$

(11) $$\begin{array}{c} Bias=\frac{a+b}{a+c} \end{array}$$

## 3. Results

### 3.1. Characteristics of Twitter Data

Examples of heat wave related tweets are given in Table 2 and Table 3. Table 2 shows tweets containing the phrase “heat wave India” in January 2015, as an example of tweets that are not related to real-time heat wave events in India (as January is a month of lower temperatures).

Table 3 shows a small extract of the tweets containing the same phrase in April 2015 as an example of tweets likely related to an event happening in real-time, as April and May 2015 experienced one of the worst recent heat waves in India [61].

**Figure 3:**
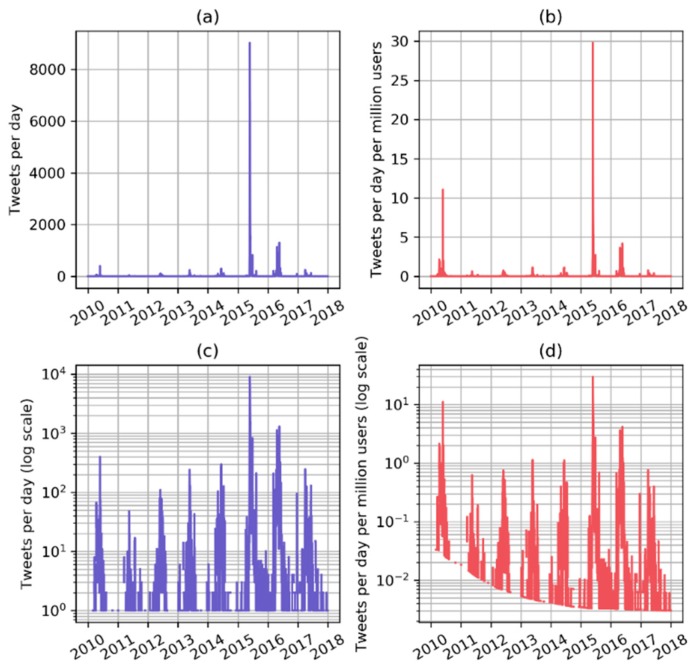
Time series of daily tweets regarding heat waves in India are plotted in panel (**a**) and in panel (**c**) using a logarithmic scale. In panel (**b**) time series of daily tweets are scaled by the number of Twitter users globally and the same time series is plotted in panel (**d**) in logarithmic scale.

**Figure 4:**
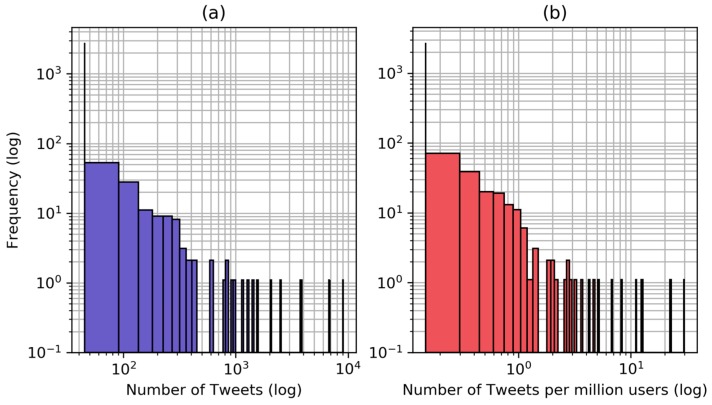
Histograms representing the distribution of the number of tweets per day (**a**) and the number of tweets per day per million users (**b**).

We count the number of tweets containing the phrase “heat wave India” or “heatwave India” each day to obtain a quantitative indicator (Figure 3). The time series have an exponential behaviour, which persists even after scaling by the global number of active Twitter users. Figure 4 shows the histograms of the number of tweets per day and of the number of tweets per day per million Twitter users in a log-log scale and the histograms show an almost linear behaviour.

### 3.2. Comparison with Mortality Data

**Figure 5:**
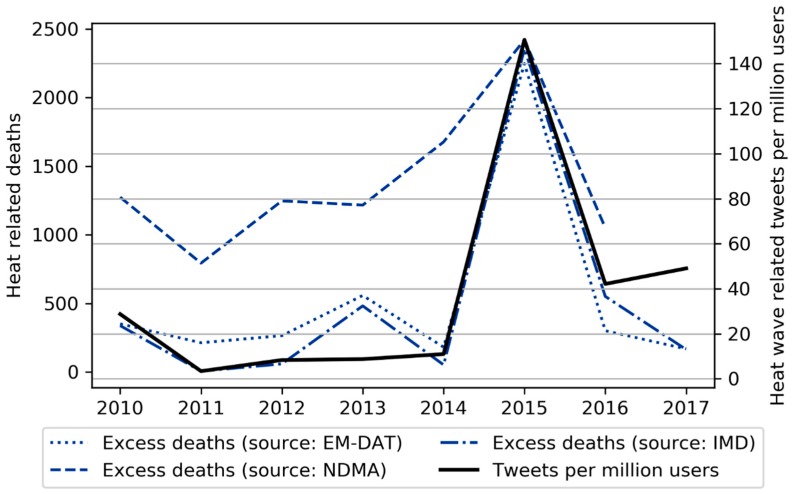
The annual number of heat wave related tweets per million users compared to three heat related mortality datasets.

The number of tweets per year containing the phrases “heat wave India” and “heatwave India” scaled by the number of active Twitter users shows a significant correlation with the three heat-related mortality datasets (Figure 5). Three mortality datasets (EM-DAT, National Disaster Management Authority—NDMA, and India Meteorological Department—IMD), are used to better account for the uncertainty in heat-related excess death information. The Pearson and the Spearman’s ranking correlation coefficients, measuring the degree of linear and monotonic relationship respectively, are calculated between the mortality data, the tweets, and the nine climate-based heat wave indicators presented in Section 2.2 are compared as well. The correlation between heat-related tweets and mortality is much stronger than between mortality and the climate-based indicators (Table 4). Twitter interest captures the extraordinary 2015 heat wave particularly well. We note also that this outlier does not overly affect the correlation, as it remains significant if the Spearman’s Rank coefficient (which is more robust to outliers) is used instead (Table 5).

The number of tweets scaled with the number of Twitter users is the only dataset that consistently reaches a significantly high correlation with all the three mortality datasets, both in terms of Pearson and Spearman’s ranking correlation coefficient (significance above 0.9 for both correlation coefficients and for all mortality datasets). Given the small number of samples and the uncertainty in the mortality data, some of the other indicators also reach high correlation values with good significance, but are not consistent throughout the three mortality datasets and score lower correlation and significance values than the Twitter dataset (see for example $T_{mM}$ in Table 4 or $T_{diffM}$ and $HI_{diffMpop}$ in Table 5). Our results therefore indicate that Twitter data is a better indicator of aggregate heat mortality than climatic indicators.

**Figure 6:**
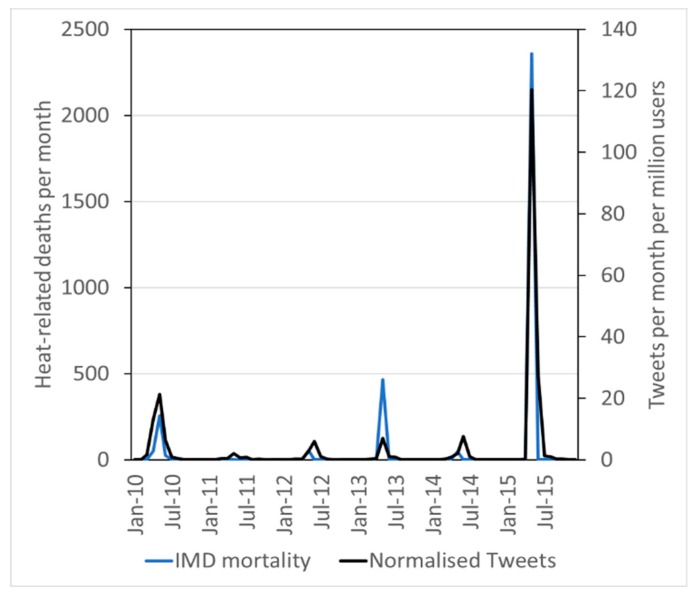
Number of tweets about heat waves in India per million users per month and number of heat-related deaths per month.

Mortality data from the EM-DAT and the NDMA datasets are only available at yearly scale. However, the IMD reports account for mortality at monthly scale too. The Pearson correlation coefficient between the number of Tweets per month per million user and the heat-related mortality per month is 0.96, while the Spearman’s ranking correlation coefficient is 0.56 (both with significance >0.99), showing that the correlation holds at monthly scale as well. Figure 6 shows how the number of tweets per million user and the number of heat-related deaths compare at monthly scale.

### 3.3. Regional Comparison with Mortality Data

The same analysis is repeated for the states of Andhra Pradesh and Telangana. Results are reported in Table 6 and Table 7.

### 3.4. Evaluation of Climatic Heat Wave Definitions

We have shown that heat wave related tweets are better correlated to heat-related mortality than other climatic indicators. Nevertheless, the use of climatic heat wave definitions is necessary, as weather data usually have longer historical records, at finer spatio-temporal resolution and allow for forecasting. Because mortality data are limited to the annual timescale, we investigate impactful heat wave detection by climate-based definitions using the daily twitter series as our reference heat wave series. This decision reflects the established strong correlation between mortality and heat wave tweets, which we assume can also identify impactful heat episodes at the daily timescale. We therefore converted the tweets to a binary series of heat wave occurrence based on a threshold of two heat wave tweets per day. Other thresholds were tested as well, but resulted in lower correlation with climate-based heat wave definitions.

These definitions are chosen either because of their relevance for the case study (IMD), or because of their popularity in literature (T95, HI95, and EHF). Contrary to the continuous climatic indicators considered for comparison with mortality, heat wave definitions are binary, i.e., differentiate between heat wave and no heat wave occurrence. For parity in our use of Twitter data as a reference we consider a heat wave day when at least two tweets about heat waves in India occur in the same day. The verification problem is of binary nature (heat wave/no heat wave), and five binary skill scores are used: Percentage Correct, Hit Rate, Miss Rate, False Alarm Rate, and Bias. The skill scores are calculated for each of the climate-based definitions and reported in Table 8.

The calculated scores provide information on different aspects of the heat wave/no heat wave distribution identified with the different definitions. The Bias shows that all the definitions significantly over estimate the number of heat wave days, of 1.74 to 2.00 times the number of days identified as heat waves with Twitter. This has an effect on the False Alarm Rate, which is very high (0.4 to 0.52). However, not all the days identified as heat waves by Twitter are identified as heat waves by the other definitions, resulting in a Hit Rate between 0.86 and 0.90 (and a complimentary Miss Rate of 0.10 to 0.14). This overestimation of heat wave days is confirmed by the ratio between the number of heat wave days and the total number of days between 2010 and 2017 (Table 9).

## 4. Discussion

At first, we look at the tweets containing the phrases “heat wave India” or “heatwave India”. The occurrence of such tweets when no real-time heat wave event happens is rare and isolated in time, while the tweets referring to an event happening in real-time are much more frequent and tend to be re-tweeted multiple times (as shown by the repetition of the same text in multiple tweets). The tweets referring to a real-time heat wave event in India seem to be mostly from news channels or informative webpages (web links in Table 3), confirming that Twitter can be used as a proxy for news media [34,35,62]. This is also due to the use of the keywords “heat wave” and “heatwave”, which is more formal than other popular expressions. However, when an event occurs, re-tweets, presumably mostly from the general public, constitute the majority of tweets, which suggests that Twitter can capture the reaction of the affected population. The tweet containing the string “Mercury crosses 40-degrees Celsius mark in north India”, shown 6 times in Table 3, is actually re-tweeted 38 times on the 19th April alone and during the peak of the 2015 heat wave the count of heat wave related tweets reached more than 9000 tweets in one day on the 26th May 2015. Most of the tweets also contain a link to a webpage, where more information about the context can be retrieved.

The number of tweets containing the heat wave related phrases is the main indicator used in this work and is scaled by the number of active Twitter users over time to account for the different tweeting base. The number of users active worldwide is used because we do not limit the origin of tweets geographically. Twitter is used by around 30 million people in India, about 2.2% of the population [63]. However, we observed that many of the tweets are from media sources and other informative channels, which report significant events in the whole country. Furthermore, it is reasonable to assume that Twitter users are concentrated in cities, where about 40% of the Indian population lives, thus making the tweets relevant for a larger share of the population. Even after being scaled by the number of users, the number of tweets per day have an power law behaviour, probably due to the occurrence of re-tweets and strongly non-linear popularity dynamics [64,65] (Figure 3 and Figure 4). This might be one of the reasons why annual Twitter data correlate better than temperature indicators with mortality data, suggesting that heat related mortality could have an exponential behaviour, as no significant adverse health effects are expected up to certain temperatures and then the impact on health gets exponentially worse for any increase in temperature or heat wave duration [66,67,68].

The comparison with some yearly temperature indicators in Table 4 and Table 5 highlights the reason why a simple quantitative definition of heat wave is not easy to find: although temperature is the trigger for heat-related mortality by definition, it is not easy to identify one single indicator that summarises the heat threat to human health, but rather many factors (maximum daily temperature, minimum night temperature, duration of the extreme weather, indoor/outdoor conditions, humidity, adaptation, and more) play a role. While the climatic heat wave definitions try to identify heat waves using a combination of causes (it is hot, thus people feel uncomfortable), the Twitter-based heat wave identification detects heat waves using an effect (people feel uncomfortable, thus they tweet about it), which makes the nature of the datasets very different. For this reason, it is expected that the tweet indicator implicitly takes into consideration a multitude of complex effects (e.g., adaptation—people start tweeting less about heat wave as they get adapted to certain heat conditions).

The strong correlation between the number of tweets per million users and the number of heat-related deaths holds at monthly scale as well, suggesting that the relationship is strong at finer temporal scale as well.

Repeating the analysis at regional level on the two states of Andhra Pradesh and Telangana confirms the good ability of Twitter to capture the consequences of heat waves, in this case represented by heat-related mortality. In particular, a very high Pearson correlation coefficient is observed, which may be skewed by the 2015 event, but a high Spearman’s ranking coefficient is observed as well, less affected by outliers. However, a better performance of climatic indicators is observed at regional level, in particular of the HI indicator, which seems to capture well the causality of heat waves and their consequences in the studied regions. Indeed, the two most affected states are geographically proximal, as shown in Figure 2, thus the climatic dynamics causing mortal heat waves are similar and more uniform, therefore easier to summarise in one climatic indicator. The South-East of India is more tropical and humid than other heatwave-prone states like Maharashtra, Madhya Pradesh, Uttar Pradesh or Gujarat. This means that the Heat Index, considering humidity as well as temperature, does well for Andhra Pradesh and Telangana, but is not able to capture all the different climatic dynamics causing uncomfortably hot conditions in India. Twitter, instead, seems to be able to capture heat-related mortality trends particularly well at medium-large scales, and therefore its added values lies in particular at larger scales.

Twitter-based heat wave identification shows very different characteristics compared to the climatic heat wave definitions. To start, notice the ratio between heat wave days and total days (Table 9): it is striking that the climatic heat wave definitions identify a heat wave occurring in the Indian subcontinent almost 2 days out of 3, while Twitter identifies only 1 out of 3. Although they look like high figures, there are two aspects that need to be considered: (1) in this work a day is considered as a heat wave day if even only 1 location in India, a country of 3.3 million km^2^, is experiencing heat wave conditions; (2) all the climatic definitions are based on climatological statistics (mean or percentiles) that are calculated over the previous 30-year period and are hence not adjusted to warming in current climate. Several studies have already observed climatic changes on the Indian climate, with increases in mean and extreme temperatures [69,70], but also an increase in heat wave frequency [44,45] and our results are consistent with this trend. However, it is still noteworthy that Twitter identifies only around half the heat wave days compared to climatic definitions (Table 9). This suggests that, while climatic definitions are more conservative, which is a good characteristic for forecasts and warnings (i.e., it is better to have a false alarm than a missed warning), Twitter can better identify the days that are uncomfortable to the population. It must also be considered that we used an empirical two-tweet threshold for the binary Twitter time series: if we used a higher threshold, it would have resulted in an even larger difference between Twitter heat wave identification and climatic heat wave definitions.

Finally, four different climatic heat wave definitions are compared. The definition by IMD has the best hit rate (and complementarily the lowest miss rate). The T95 definition and the EHF definitions have a lower bias (−11% and −12% respectively, compared to IMD), resulting in a lower false alarm rate (−18% and −20% respectively) and higher percentage correct value (+8% and +10% respectively), which denotes less over-estimation of heat wave days, but have a lower hit rate (−3% and −2% respectively), which denotes less precision. The differences between the T95 and the EHF scores are minimal, but the EHF bias is slightly lower (−2%), suggesting that considering cumulative short-term effects and seasonal adaptation has a small, but observable effect. Finally, the HI95 definition, based on the Heat Index and thus corrected for humidity performs a little worse, having both high bias and low hit rate. The reason why the Heat Index is not performing as well as other indices is due to the fact that heat waves occur during the dry season in India, when humidity is low. Our results provide confidence in the IMD definition, which is operationally used to issue warnings. The high bias shows that, although lacking some precision, the definition is conservative and issues warnings in most potentially dangerous situations, while the high hit rate suggests precision in identifying heat waves in the Indian climate.

However, it is clear that, despite the differences between them, the climate-based definitions produce results more similar to each other than the heat wave identification method based on Twitter. The conservative nature of the climatic heat wave definitions, the possibility to be applied to future weather forecasts, and the availability of weather data at fine spatio-temporal scale makes the use of climatic heat wave definitions particularly well-suited to issue warnings and forecasts. Additionally, the availability of long time series of weather data allows the use of climate-based definitions when considering heat wave trends and climate change. However, Twitter showed significantly better performance in estimating heat related mortality that is not addressed by climatic heat wave definitions. More complex heat wave definitions considering climatic, socioeconomic and demographic factors could have performed better in capturing heat-related mortality, but it would reinforce the concept that heat-related mortality is not captured by any simple climatic indicator, but by a complex combination of factors. The Twitter signal and the climatic definitions can, therefore, be seen as complementary to one another.

The presented work is the first analysing the use of Twitter for heat wave identification at large geographical scale and as such it is more of a proof of concept rather than an exhaustive analysis of the methodology’s advantages and disadvantages. India has been selected as a case study as it is a large developing country strongly affected by heat waves, but only limited and uncertain data was available for the analysis. Following work will need to verify how Twitter works to identify heat waves in different contexts and with more data, maybe using other languages than English. The use of Twitter data to identify heat waves is used as a proxy for heat wave mortality and morbidity, and as such it has a limitations: (1) only a part of the population uses Twitter and usually not the most affected portion (elderly, infants, homeless); (2) the selected keywords do not capture all the tweets about heat waves and not all the identified tweets are indeed about real time heat waves; (3) social media data is affected by popularity of certain topics and complex social dynamics, which may vary the frequency of heat wave related tweets independently on the heat wave occurrence. However, in spite of these approximations the proxy still shows a significant ability to identify dangerous heat wave events and therefore can be very useful.

A real-time tracking of heat wave related tweets can be used to identify real-time discomfort conditions, prioritising any response in space and time. This has already been used successfully for earthquake response for example [38], and our results suggest that a similar approach could be used to identify dangerous heat waves in real time as well. Additionally, heat wave related tweets can also be used as a time series of past heat waves, identifying impactful events to improve understanding of event drivers [71] and facilitate targeted adaptation [72]. This would be particularly, useful in developing countries where mortality data are scarce, difficult to access, and very uncertain, and for studies at larger scales. Indeed, one of the biggest limitations of heat wave impact studies so far is that they usually cannot address large (national/international) scales, as mortality data are available only at hospital/community level. Twitter data could help fill this gap in research.

## 5. Conclusions

This study has shown that the number of tweets about heat waves in India has a strong correlation with the number of heat-related excess deaths, holding through spatial and temporal scales, and that Twitter is more precise in identifying heat wave events that have an impact on the population. This is a precious resources that should not be seen as a replacement of climatic data, but rather an integration.

We see two primary role of Twitter data for heat wave identification. On the one hand, Twitter can be used in real time to detect heat wave events that are dangerous for the population, in particular at larger geographical scales. On the other hand, the scientific community is working hard to identify quantitative heat wave definitions able to capture the health effects of heat, but a great challenge is the lack of consistent, large scale, heat-related mortality or morbidity datasets. Several studies in several different communities result in different conclusions about the best heat wave definition in this regard. Twitter data, although less precise than hospital records, are available worldwide, are much more easily accessible, and could make the difference in identifying heat wave definitions that are effective in capturing heat impact on health globally. Although this study only analyses India and availability of data is limited, the conclusions could be relevant for other developing countries and more work needs to be done to evaluate Twitter strengths and limitations in a heat wave identification context. It is clear from this work, that the use of social media in identifying heat waves holds considerable promise for increasing societal resilience to the growing challenges posed by extreme heat.

## Figures and Tables

**Table 1:** Number of heat-related deaths per year per Indian state according to IMD weather reports.

State	2010	2011	2012	2013	2014	2015	Total
Andhra Pradesh	75	0	5	442	0	1722	2244
Telangana	0	0	0	0	0	585	585
Maharashtra	158	1	1	5	0	0	165
Odisha	33	0	30	4	47	41	155
West Bengal	18	0	11	5	4	0	38
Jharkhand	19	0	7	6	0	0	32
Chattisgarh	9	0	3	0	0	0	12
Madhya Pradesh	9	0	0	3	0	0	12
Kerala	2	3	0	3	0	0	8
Uttar Pradesh	3	0	0	5	0	0	8
Gujarat	1	0	0	0	0	7	8
Punjab	0	0	0	6	0	0	6
Haryana	0	0	0	2	0	2	4
Rajasthan	7	0	0	0	0	0	7
Bihran	3	0	0	0	0	0	3
Chandigarh	0	1	1	0	0	0	2

**Table 2:** Example Tweets containing the phrase “heat wave India” in January 2015.

2015-01-29 04:41:09	@ corrado_19 @ PatrickGorman3 # Rejected Yankee Candles India during a heat wave
2015-01-28 12:30:35	5 June 2003—A severe heat wave across Pakistan and India reaches its peak, as temperatures exceed 50 °C (122 °F) in the region
2015-01-23 09:15:34	5 June 2003—A severe heat wave across Pakistan and India reaches its peak, as temperatures exceed 50 °C (122 °F) in the region
2015-01-18 19:04:11	Wet shoes like the Southern India heat wave of 2003, leading to the deaths of 1500.
2015-01-12 22:00:48	A bent phone a bit like the Southern India heat wave of 2003 which killed 1500.

**Table 3:** Some of the tweets containing the phrase “heat wave India” in April 2015. The heat wave of 2015 started in April and reached its peak in May.

2015-04-19 18:28:06	Mercury crosses 40-degrees celsius mark in north India: Heat wave-like conditions prevailed at several places … http://bit.ly/1cMar4X
2015-04-19 18:33:10	Check this @ SuryaRay Mercury crosses 40-degrees celsius mark in north India: Heat wave-like … http://dlvr.it/9SZKBK#SuryaRay#India
2015-04-19 18:46:57	Mercury crosses 40 °C mark in north India: Heat wave-like conditions prevailed at several places across the cou … http://bit.ly/1DpuPOL
2015-04-19 19:05:45	Mercury crosses 40-degrees celsius mark in north India—Heat wave-like conditions prevailed at several places acr … http://ow.ly/2XAYX9
2015-04-19 19:21:47	RT- Mercury crosses 40-degrees celsius mark in north India: Heat wave-like conditions prevailed at sever … http://bit.ly/1DpKIox#News
2015-04-19 19:21:51	Mercury crosses 40-degrees celsius mark in north India: Heat wave-like conditions prevailed at several places … http://bit.ly/1F2bfhg
2015-04-20 03:41:16	Mercury crosses 40-degrees celsius mark in North India: Heat wave-like conditions prevailed at several places … http://bit.ly/1DrnY7J
2015-04-21 10:14:57	MET DEPARTMENT WARNS OF HEAT WAVE IN WESTERN INDIA. Ahmedabad temp. can be max 44* Be aware have full water, cover face & head.
2015-04-30 04:19:29	India, Asia at Thu, 30 April 2015 03:19:28 +0000|# Heat Wave event has been observed in India, Asia|http://bit.ly/1zrQ1sk
2015-04-30 04:24:41	# incident: Heat Wave—Asia—India: 30.04.2015—03:18:46—Heat Wave event happened in Asia/India. http://bit.ly/1bWJpYu
2015-04-30 04:24:43	Heat Wave—Asia—India http://bit.ly/1bWJpYu 4moInfoClkDescrption
2015-04-30 04:26:05	Heat Wave—Asia—India: 30.04.2015—03:18:46—Heat Wave event happened in Asia/India. http://dlvr.it/9c4B1q vía @ RSOE_EDIS
2015-04-30 04:26:06	# RSOE_EDIS Heat Wave—Asia—India http://dlvr.it/9c4DyY
2015-04-30 04:27:07	Heat Wave—Asia—India|Details: http://ift.tt/1ETge5e
2015-04-30 04:27:32	Reporte: RSOE-EDIS Heat Wave—Asia—India http://ift.tt/1ETge5e
2015-04-30 05:00:07	Heat Wave—Asia—India http://dlvr.it/9c57pG

**Table 4:** Pearson correlation coefficient between mortality data according to the three available mortality databases and the Twitter and heat wave indicators, together with their significance (1 − *p*).

	Pearson Correlation Coefficient			Significance		
	EM-DAT	NDMA	IMD	EM-DAT	NDMA	IMD
Twitter	0.94	0.97	0.82	>0.99	>0.99	0.98
$T_{mM}$	−0.62	−0.49	−0.70	0.90	0.78	0.92
$T_{mM}$	−0.36	−0.27	−0.31	0.62	0.47	0.50
${\mathrm{HI}}_{M}$	0.11	0.03	0.16	0.20	0.06	0.26
${\mathrm{EHF}}_{M}$	0.07	0.15	0.06	0.13	0.27	0.11
$T_{\mathrm{diffM}}$	0.05	0.15	−0.10	0.09	0.27	0.16
${\mathrm{HI}}_{\mathrm{diffM}}$	0.08	−0.07	0.13	0.14	0.13	0.22
$T_{\mathrm{diffMpop}}$	−0.01	0.07	0.16	0.02	0.13	0.27
${\mathrm{HI}}_{\mathrm{diffMpop}}$	0.11	0.27	0.22	0.20	0.49	0.37
${\mathrm{EHF}}_{\mathrm{Mpop}}$	0.12	0.19	0.28	0.22	0.35	0.46

**Table 5:** Spearman’s ranking correlation coefficient between mortality data according to the three available mortality databases and the considered Twitter and climatic heat wave indicators, together with their significance.

	Spearman’s Ranking Correlation Coefficient			Significance		
	EM-DAT	NDMA	IMD	EM-DAT	NDMA	IMD
Twitter	0.62	0.67	0.71	0.90	0.93	0.93
$T_{\mathrm{mM}}$	−0.43	−0.29	−0.64	0.71	0.51	0.88
$T_{\mathrm{MM}}$	−0.29	0.00	−0.18	0.51	0.00	0.30
${\mathrm{HI}}_{M}$	−0.19	−0.19	−0.14	0.35	0.35	0.24
${\mathrm{EHF}}_{M}$	0.19	0.19	0.00	0.35	0.35	0.00
$T_{\mathrm{diffM}}$	0.19	0.48	−0.14	0.35	0.77	0.24
${\mathrm{HI}}_{\mathrm{diffM}}$	−0.12	−0.29	−0.11	0.22	0.51	0.18
$T_{\mathrm{diffMpop}}$	0.05	0.24	0.32	0.09	0.43	0.52
${\mathrm{HI}}_{\mathrm{diffMpop}}$	0.00	0.60	0.43	0.00	0.88	0.66
${\mathrm{EHF}}_{\mathrm{Mpop}}$	0.21	0.33	0.21	0.39	0.58	0.36

**Table 6:** Pearson correlation coefficient between IMD mortality data, Twitter and heat wave indicators, together with their significance (1 − *p*) for Andhra Pradesh and Telangana.

	Andhra Pradesh	Telangana	Andhra Pradesh	Telangana
Twitter	0.97	>0.99	>0.99	>0.99
$T_{\mathrm{mM}}$	0.57	0.22	0.76	0.32
$T_{\mathrm{MM}}$	−0.31	−0.27	0.45	0.40
${\mathrm{HI}}_{M}$	0.55	0.77	0.74	0.93
${\mathrm{EHF}}_{M}$	0.48	0.37	0.66	0.53
$T_{\mathrm{diffM}}$	0.26	0.25	0.39	0.37
${\mathrm{HI}}_{\mathrm{diffM}}$	0.61	0.79	0.80	0.94
$T_{\mathrm{diffMpop}}$	−0.05	−0.04	0.07	0.07
${\mathrm{HI}}_{\mathrm{diffMpop}}$	0.23	0.28	0.33	0.41
${\mathrm{EHF}}_{\mathrm{Mpop}}$	0.23	0.19	0.34	0.29

**Table 7:** Spearman’s ranking coefficient between IMD mortality data, Twitter and heat wave indicators, together with their significance (1 − *p*) for Andhra Pradesh and Telangana.

	Andhra Pradesh	Telangana	Andhra Pradesh	Telangana
Twitter	0.81	0.65	0.95	0.84
$T_{\mathrm{mM}}$	0.81	0.13	0.95	0.20
$T_{\mathrm{MM}}$	−0.29	−0.13	0.42	0.20
${\mathrm{HI}}_{M}$	0.64	0.65	0.83	0.84
${\mathrm{EHF}}_{M}$	0.64	0.39	0.83	0.56
$T_{\mathrm{diffM}}$	0.43	0.39	0.61	0.56
${\mathrm{HI}}_{\mathrm{diffM}}$	0.81	0.65	0.95	0.84
$T_{\mathrm{diffMpop}}$	0.00	−0.13	0.00	0.20
${\mathrm{HI}}_{\mathrm{diffMpop}}$	0.70	0.39	0.88	0.56
${\mathrm{EHF}}_{\mathrm{Mpop}}$	0.58	0.13	0.77	0.20

**Table 8:** Skill scores evaluating the performance of the climate-based heat wave definitions against the Twitter based definition.

	IMD	T95	HI95	EHF
Percentage Correct	0.62	0.67	0.60	0.68
Hit Rate	0.90	0.87	0.86	0.86
Miss Rate	0.10	0.13	0.14	0.14
False Alarm Rate	0.51	0.42	0.52	0.41
Bias	2.00	1.78	2.00	1.74

**Table 9:** Ratio between the number of days identified as heat wave days and the total number of days considered (TW = Twitter).

	IMD	T95	HI95	EHF	TW
Absolute fraction of heat wave days	0.63	0.56	0.63	0.55	0.32
